# Supplementary material for: In operando cryo-STEM of pulse-induced charge density wave switching in TaS2
Source: Nat Commun. 2023 Dec 11;14:8202. doi: 10.1038/s41467-023-44093-2 (PMC10713631; doi:10.1038/s41467-023-44093-2)
Supplement: Supplementary file 1 — Supplementary Information [file 41467_2023_44093_MOESM1_ESM.pdf]

In operando cryo-STEM of pulse-induced charge density wave switching in TaS_2

Supplementary Information

James L Hart[1], Saif Siddique[1], Noah Schnitzer[1], Stephen D. Funni[1], Lena F. Kourkoutis[2,3], Judy J. Cha[1]

1. Department of Materials Science and Engineering, Cornell University, United States
2. School of Applied and Engineering Physics, Cornell University, United States
3. Kavli Institute at Cornell for Nanoscale Science, Cornell University, United States
*Corresponding author: jc476@cornell.edu

Supplementary Note 1: Electrical biasing equipment and set-up

For the electrical measurements shown in Figs. 1, 2, and 5 of the main text, we used a Keithley 2400 SMU to perform 2-terminal biasing. For the set-up shown in Fig. 3, we used a Keysight 33600A waveform generator to produce the voltage pulses, and we used a Tektronix DPO2024 oscilloscope to monitor the V_{flake} and V_{total} signals. For the data shown in Fig. 4 of the main text, we used a similar set-up to that shown in Fig. 3, but with an additional 30 Ω series resistor. The set-up is shown in Supplementary Figure 5. For these experiments, we applied a small DC voltage bias in addition to the pulse. By measuring the resistance drop across the 30 Ω resistor with the Keithley SMU, we are then able to monitor the flake resistance on a time-scale of several seconds. Conversely, the oscilloscope only allows measurement time-scales of several μs.

Supplementary Note 2: Diffraction pattern processing

To analyze the diffraction data, the position of each Bragg and 2nd order CDW spot is determined using a center-of-mass (COM) algorithm (we use the 2nd order CDW spots since they have a high intensity for both the C and NC phases, in contrast to the 1st order CDW spots). First, the spot position is guessed based on user input. Next, the spot position is updated to the maximum pixel intensity within a local 3 × 3 pixel window. Then, to achieve sub-pixel accuracy, the spot position is refined using COM. To implement the COM refinement, we first up-sample the data by ≈3× using linear interpolation. Then, we perform a background subtraction by fitting a plane to all pixels within the local background region, which falls between the circles of radii R_1 and R_2 as shown in Supplementary Figure 6b,c. After the background subtraction is performed, the COM is computed using all pixels within the circle of radius R_1, and the spot position is updated. This COM refinement is performed three successive times. The radii R_1 and R_2 were adjusted based on the width of the CDW peaks for each diffraction pattern, and were around 2 pixels and 3 pixels, respectively. Accounting for the up-sampling, R_1 and R_2 would be 6 and 9 pixels, respectively.

Next, for each 2nd order CDW spot, the angle φ_{2nd} and magnitude k_{2nd} are determined relative to the local Bragg diffraction vectors **a*** and **b*** (Supplementary Figure 6d). Specifically, k_{2nd} is determined by $k_{2nd} = |\mathbf{Q_{2nd}}| / ((|\mathbf{a^*}| + |\mathbf{b^*}|) / 2)$. φ_{2nd} is initially calculated as the angle between $\mathbf{Q_{2nd}}$ and **a***, and then adjusted by the factor 60° / α_{ab}, where α_{ab} is the measured angle between

the local **a*** and **b*** vectors. By defining the CDW angle φ_{2nd} and magnitude k_{2nd} relative to the local Bragg vectors, any distortions in the diffraction pattern are corrected. From the 2nd order CDW spot positions, the 1st order CDW spot positions are calculated based on simply trigonometry: $\varphi_{1st} = 30° - \varphi_{2nd}$, and $k_{1st} = k_{2nd} / (2 * \cos(30°))$.

Thus, for each CDW spot, we calculate φ and *k*. Most diffraction patterns include $N \approx 30$ CDW spots. The reported values of φ and *k* are the mean values for an entire diffraction pattern. The reported error bars (Fig. 1g-i of the main text) are the standard deviation of the measured value (either φ or *k*) divided by the square root of *N*. As an example, Supplementary Figure 6e shows the measured values of φ for the diffraction pattern shown in Supplementary Fig. 6a. Here, $N = 27$, the mean value is $\varphi = 13.87°$, the standard deviation is $\sigma = 0.30°$, and the standard error is $\sigma / N^{1/2} = 0.06°$. Having φ and *k*, we then calculate the CDW domain size D_{NC} using Equation 1 of the main text, which is copied below for reference

$$D_{NC} = a/\sqrt{(2\pi\Delta\varphi/360)^2 + (\Delta k/k_C)^2} \quad (1)$$

where *a* is the atomic lattice parameter, and Δφ and Δ*k* are the differences in φ and *k* relative to their commensurate values.

Note that the processing steps described above were used for both the SAED data collected as a function of temperature and voltage (Figs. 1, 2, and 4 of the main text), as well as the 4D-STEM mapping experiment (Fig. 5 of the main text). Supplementary Figure 7 presents additional data for the 4D-STEM mapping experiment, specifically, a full diffraction pattern is shown, as well as maps of the CDW angle φ and magnitude *k*. This dataset was collected at 120 K and at 0 V, after having applied 0.6 V which induced switching. For this experiment, a portion of the EMPAD detector was faulty (see dark pixels), and only the diffraction spots away from the defective region were used for analysis.

Supplementary Note 3: Strain measurements and coefficient of thermal expansion

To determine the flake strain, we fit the Bragg peak positions using the same COM algorithm described in Supplementary Note 2. Then, we fit all of the measured Bragg peaks to a reciprocal-space lattice defined by the reciprocal lattice vectors **a*** and **b***. The vectors **a*** and **b*** are determined using a least-squares fit. **a*** and **b*** are not orthogonal (see Supplementary Fig. 6d); thus, to obtain orthogonal strain components, we extract the component of **b*** which is perpendicular to **a***. This yields two orthogonal in-plane vectors. From these, we calculate the average in-plane strain ε. For each diffraction measurement, we set $\varepsilon = 0.0\%$ at the beginning of the voltage ramp since we are only concerned with the relative change in strain, not the absolute strain.

To determine the error in our strain measurements, we measure the flake strain for 10 s without any temperature changes or applied bias. We then take the standard deviation of the strain and obtain a standard error of $\sigma = 0.0014\%$. This represents the statistical error for relative strain measurements, assuming that there is minimal sample tilt or movement during a given measurement. Inspection of the diffraction patterns during applied bias (e.g. Supplementary Video 2) shows no changes in the Bragg peak intensities, supporting our assumption of minimal sample

tilt or movement during biasing experiments. Conversely, during CDW switching there is evidence of flake tilting (e.g. Supplementary Video 3). However, our strain (and temperature) quantification is restricted to the C phase and does not include any CDW switching. Lastly, we note that the error for measuring the absolute strain is much higher than σ = 0.0014%, but for our experiments, only the relative strain is needed.

To determine the thermal coefficient of expansion α for this TaS_2 flake, we performed heating experiments at 0.4 K / s starting at 110 K (Supplementary Figure 8). Measurements were performed before the in situ biasing experiments (Run 1) and after all of the in situ biasing experiments (Run 2). For each run, we perform a linear fit to the data from 110 – 180 K where the flake is in the C phase. From these fits, we extract $\alpha = 9.5 \pm 0.8 \times 10^{-4}$ % / K. The error of 0.8×10^{-4} % / K reflects the differing α values calculated for Runs 1 and 2. Our data is in good agreement with literature reports of bulk TaS_2[22,23], as shown in Supplementary Figure 8. We note that the measured strain occasionally jumps discontinuously, both during temperature sweeps and after application of large voltage bias (for example see Fig. 2c of the main text, 0.8 V curve, from 10 – 20 s). These jumps occur after large strain events, in particular, the C to NC phase transition. This behavior is attributed to sliding of the flake relative to the SiN_x substrate. Because these jumps occur after the C to NC phase transition, they do not influence our strain and temperature analysis, which is entirely focused on strain within the C phase prior to the CDW transition.

Supplementary Note 4: Thermal modeling of the membrane

The membrane temperature is determined as described in refs. 19 and 20. To determine the temperature difference between the flake and the membrane, ΔT, we model the temperature distribution assuming steady-state heat transfer in a system with cylindrical symmetry:

$$\Delta T = {IV \ln\left(R_{mem}/R_{flake}\right)}\big/{2\pi t\kappa} \qquad (2)$$

where the heat generated Q is determined by the applied current I and voltage V, and the heat flows radially outward from the flake. R_{mem} and R_{flake} are the radii of the Pt coil and the flake (we use 100 and 10 μm, respectively), t is the thickness of the membrane (we use 100 nm), and κ is the thermal conductivity. Using a thermal conductivity of 10 W / mK for silicon nitride, we find that $\Delta T \approx 100$ K. Hence, given our biasing conditions and a membrane temperature of 130 K, we expect the flake temperature to surpass $T_c = 200$ K, consistent with the Joule heating hypothesis.

Supplementary Figures and Videos:

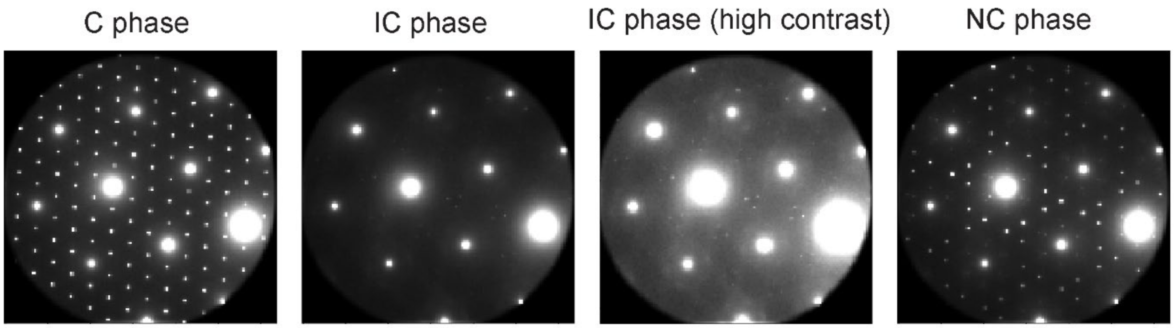

Supplementary Figure 1 | Diffraction patterns during switching. Full diffraction patterns for the triangular voltage ramp with V_{max} = 0.8 V, showing the C, IC, and NC phases. Cropped versions of these patterns are shown in the Fig. 2b inset.

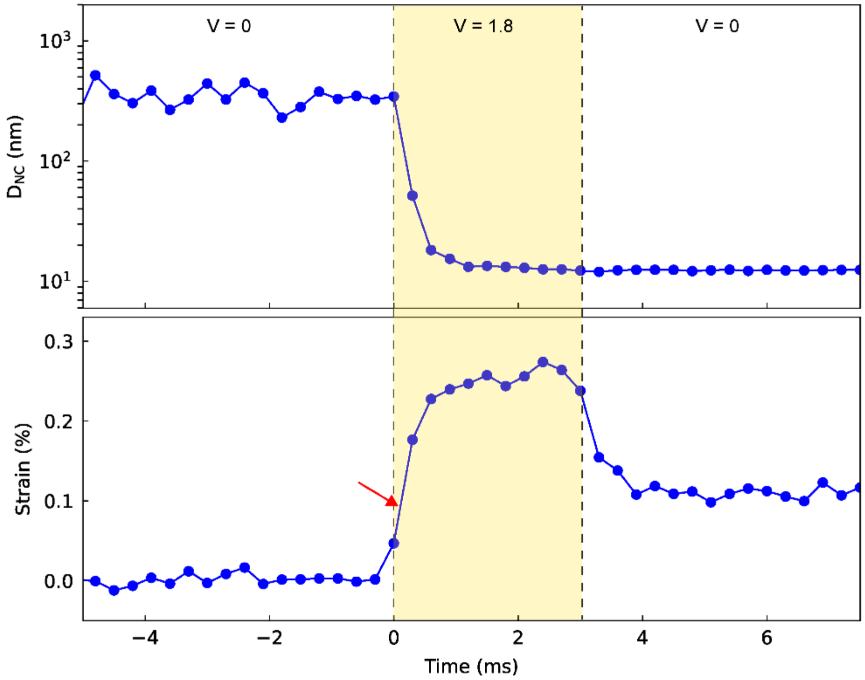

Supplementary Figure 2 | CDW structure and strain during pulsing. Time-resolved comparison of the CDW domain size D_{NC} and the in-plane strain ε during a square 1.8 V pulse with a 3 ms duration at a temperature of 110 K (Fig. 3 of the main text). Time resolution is 300 μs. At time t = 0, there is no change in the CDW structure; however, there is a strain of 0.06%, indicating a temperature rise to ≈173 K. The next datapoint indicates that the temperature has surpassed T_c ≈ 200 K, and the flake transitions from the C to the NC phase. The red arrows marks ε = 0.09% which indicates T = T_c. After the 3 ms pulse is complete, the strain quickly relaxes from ≈0.25% to 0.1%. This remnant strain is attributed to CDW-lattice coupling across the C to NC phase transition. The EMPAD and pulse generator are not time-synced; we manually set t = 0 as the first datapoint to show a deviation in the lattice strain.

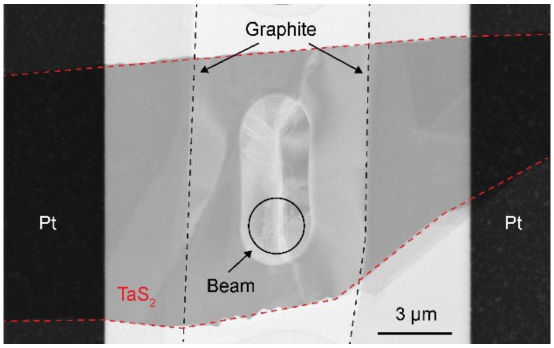

Supplementary Figure 3 | Device overview. STEM-HAADF image of the TaS_2 device, with the beam position for SAED shown.

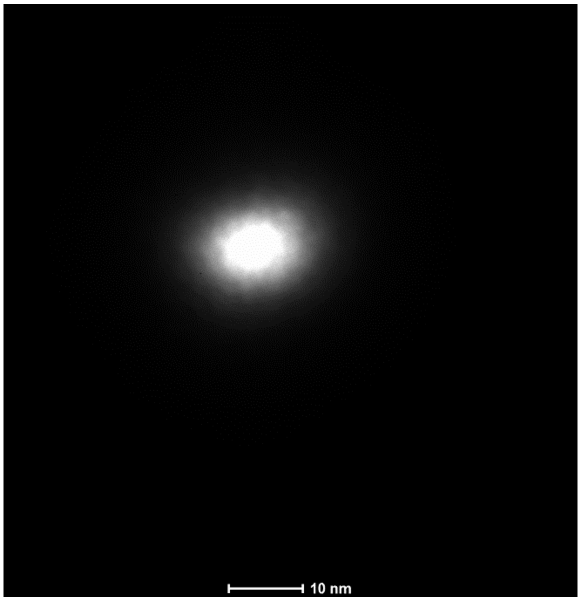

Supplementary Figure 4 | STEM probe. Real space image of the STEM probe used for 4D-STEM mapping, with a nominal convergence angle of 0.15 mrad.

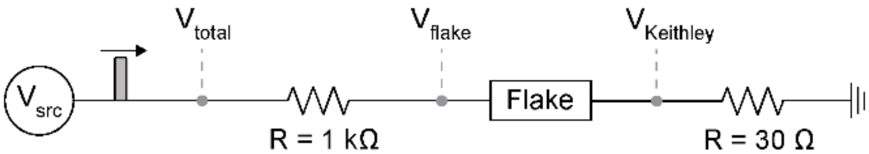

Supplementary Figure 5 | Biasing set-up. The biasing set-up used for Figure 4 of the main text. V_{total} and V_{flake} are measured with the oscilloscope, and $V_{Keithley}$ is measured with the Keithley SMU.

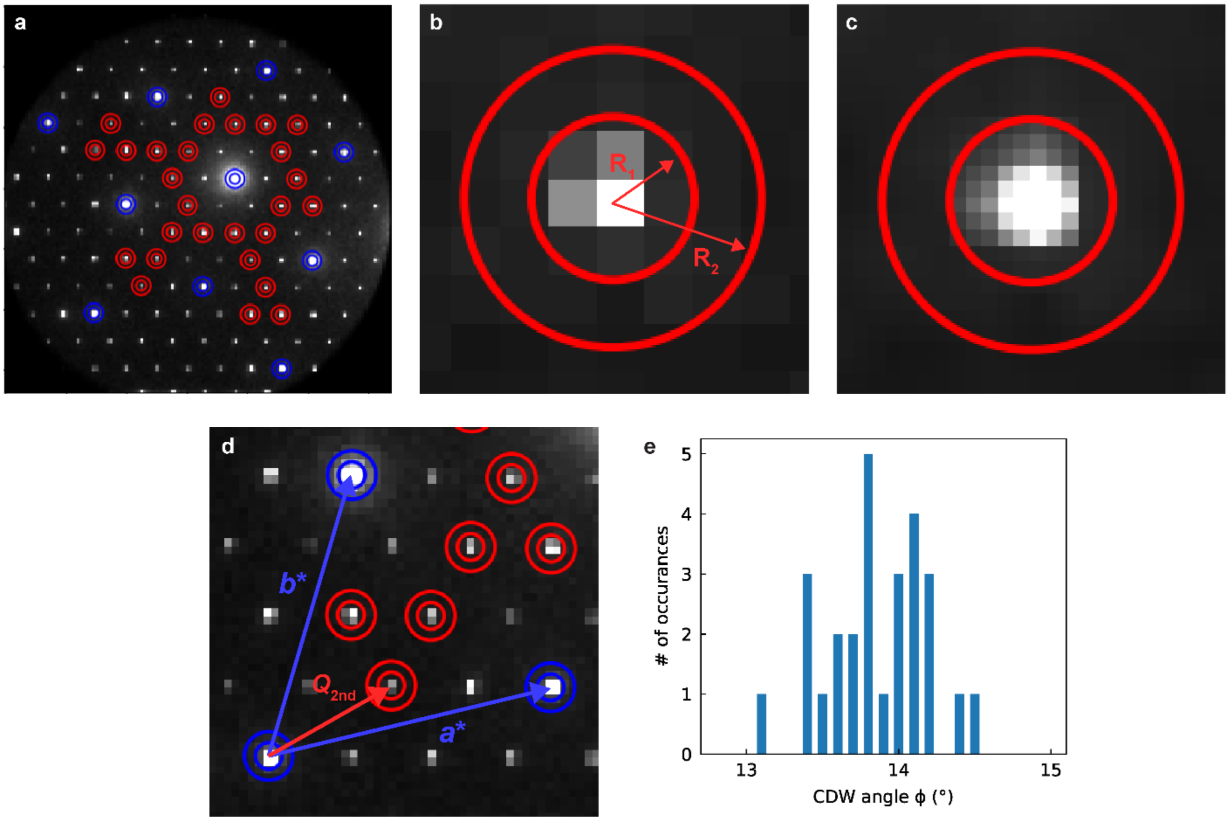

Supplementary Figure 6 | COM analysis. a. Example diffraction pattern captured on the EMPAD. The processed Bragg and CDW spots are circled in blue and red, respectively. For each spot, the inner circle defines the area used for COM refinement, and the outer circle defines the area used for background subtraction prior to the COM refinement. **b.** Zoomed in view of a CDW spot, showing the R_1 and R_2 vectors used for COM refinement and background subtraction (see Supplementary Note 2). **c.** Same as **b**, but up-sampled by 3x, as implemented in our COM refinement. **d.** Zoomed in region showing the **Q** vector for a 2nd order CDW spot, as well as the local Bragg diffraction vectors. **e.** Histogram of all the CDW angles extracted from the diffraction pattern shown in **a**, with N = 27, a mean angle of φ = 13.87°, a standard deviation of σ = 0.30°, and a standard error of $\sigma / N^{1/2}$ = 0.06°.

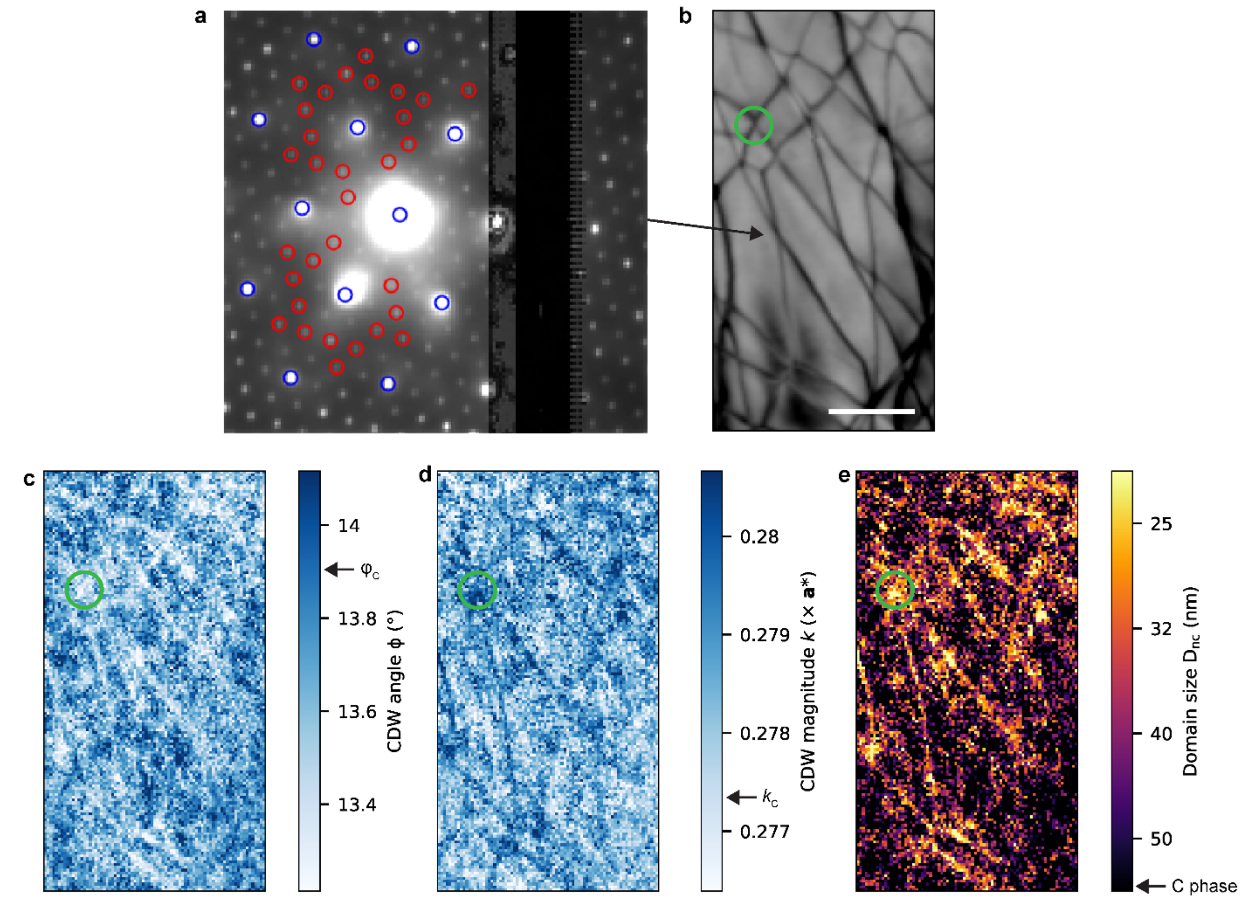

Supplementary Figure 7 | 4D-STEM CDW mapping. a. Extracted diffraction pattern from the 4D-STEM mapping experiment. The black region on the right side of the image corresponds to defective pixels. Bragg peaks are circled in blue, and CDW peaks in red. Note that the central Bragg peak has a full width at half-maximum of ~1.5 pixels, and the detector is not saturated. However, the contrast limits (chosen to highlight the CDW spots) make the peak appear much broader. **b.** Virtual STEM image, showing a network of basal dislocations. The scale bar is 1 μm and applies to **c**-**e** as well. **c.** Map of the CDW angle φ. **d.** Map of the CDW magnitude *k*. **e.** Map of the CDW domain size, D_{NC}, generated from the φ and *k* data and Equation 1. The green circles in **b**-**e** mark the same region of the sample, which shows an intersection of dislocations (**b**), a local decrease in the CDW angle consistent with the NC phase (**c**), a local increase in the CDW vector magnitude *k* also consistent with the NC phase (**d**), and lastly a large decrease in the domain size D_{NC} (**e**).

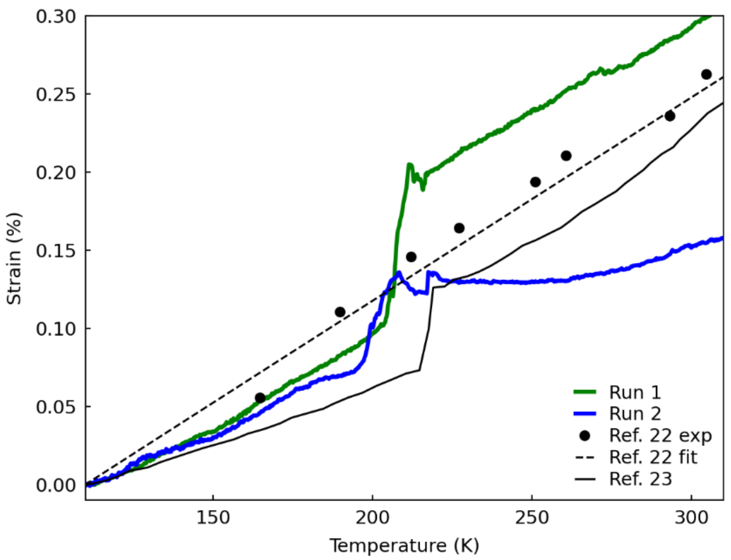

Supplementary Figure 8 | Strain vs. temperature. In-plane strain versus temperature for our TaS_2 device measured within the TEM (Runs 1 and 2), as well as data taken from the literature. The data from ref. 23 is from a capacitance dilatometer, and the data from ref. 22 is from X-ray diffraction. The ref. 22 fit is a linear fit to experimental data from 170 to 500 K; we extended the fit line down to 110 K for comparison to our experiments.
