# Supplementary material for: In operando cryo-STEM of pulse-induced charge density wave switching in TaS2
Source: Nat Commun. 2023 Dec 11;14:8202. doi: 10.1038/s41467-023-44093-2 (PMC10713631; doi:10.1038/s41467-023-44093-2)
Supplement: Supplementary file 3 — Description of Additional Supplementary Files [file 41467_2023_44093_MOESM3_ESM.pdf]

Description of Additional Supplementary Files

Supplementary Movie 1 | Diffraction video during heating of TaS2 from the C phase to the NC phase. This video corresponds to the data in Fig. 1 of the main text.

Supplementary Movie 2 | Diffraction video during a 0.7 V ramp, corresponding to the data shown in Fig.2 of the main text.

Supplementary Movie 3 | Diffraction video during a 0.8 V ramp, corresponding to the data shown in Fig.2 of the main text.

Supplementary Movie 4 | Diffraction video during a 1.8 V pulse with a 3 ms duration, taken at 110 K. This video corresponds to the data in Fig. 3b and 3c of the main text.

Supplementary Movie 5 | Diffraction video during a 9.6 V pulse with a 3 μs duration, taken at 110 K. This video corresponds to the data in Fig. 4 of the main text.
